# Evolution and current state of global research on paediatric resuscitation: a systematic scientometric analysis

**DOI:** 10.1186/s13049-020-00780-3

**Published:** 2020-09-10

**Authors:** Sean S. Scholz, Rainer Borgstedt, Leoni C. Menzel, Sebastian Rehberg, Gerrit Jansen

**Affiliations:** 1 Department of Anaesthesiology, Intensive Care, Emergency Medicine, Transfusion Medicine and Pain Therapy, Protestant Hospital of the Bethel Foundation, Burgsteig 13, Haus Gilead I, 33617 Bielefeld, Germany; 2 Institute for Diagnostic and Interventional Radiology, Protestant Hospital of the Bethel Foundation, Bielefeld, Germany

**Keywords:** Cardiopulmonary resuscitation, Pediatric, Gender disparities, Critical care, Infant mortality

## Abstract

**Background:**

Paediatric resuscitation is rare but potentially associated with maximal lifetime reduction. Notably, several nations experience high infant mortality rates even today. To improve clinical outcomes and promote research, detailed analyses on evolution and current state of research on paediatric resuscitation are necessary.

**Methods:**

Research on paediatric resuscitation published in-between 1900 and 2019 were searched using Web of Science. Metadata were extracted and analyzed based on the science performance evaluation (SciPE) protocol. Research performance was evaluated regarding quality and quantity over time, including comparisons to adult resuscitation. National research performance was related to population, financial capacities, infant mortality rate, collaborations, and authors’ gender.

**Results:**

Similar to adult resuscitation, research performance on paediatric resuscitation grew exponentially with most original articles being published during the last decade (1106/1896). The absolute number, however, is only 14% compared to adults. The United States dominate global research by contributing the highest number of articles (777), Hirsch-Index (70), and citations (18,863). The most productive collaboration was between the United States and Canada (52). When considering nation’s population and gross domestic product (GDP) rate, Norway is leading regarding population per article (62,467), per Hirsch-Index (223,841), per citation (2226), and per GDP (2.3E-04). Regarding publications per infant mortality rate, efforts of India and Brazil are remarkable. Out of the 100 most frequently publishing researchers, 25% were female.

**Conclusion:**

Research efforts on paediatric resuscitation have increased but remain underrepresented. Specifically, nations with high infant mortality rates should be integrated by collaborations. Additional efforts are required to overcome gender disparities.

## Introduction

It is essential to obtain the best possible scientific evidence, particularly in paediatric resuscitation, where a maximum of potential lifetime can be gained or lost [1, 2]. If the annual approximate number of in- and out of hospital cardiac arrests are combined, there are about 22,000 cardiac arrests in children in the United States; compared to 640,000 in adults [1–3]. Importantly, the mere incidence of events regarding a highly developed health care setting does not outweigh the great relevance of this topic. This is illustrated by globally significantly diverging infant mortality rates (deaths per 1000 life births) ranging from high infant mortality rates in Afghanistan (104) or various African countries, such as Somalia (90), to particularly low rates in the United states (5) or European countries, e.g. Germany, France, and Italy, where infant mortality rates are below 4 [4]. One of the difficulties in research on paediatric resuscitation are the heterogeneities of the patient collective (e.g. age, underlying disease) and researching nations (financial setting, population), which complicate interpretation of the results. Additionally, studies on paediatric resuscitation are limited due to their long observational period and relatively rare events [3–6]. For instance, the prospective observational multicenter APRICOT study included 30,847 children undergoing anesthesiologic procedures in 261 centers across 33 European countries but documented only 8 cardiac arrests [7]. Despite its great relevance, international research efforts on paediatric resuscitation have not been systematically evaluated. Therefore, we aimed at assessing global research on paediatric resuscitation to analyze its architecture and to further ignite research efforts [8]. In addition to absolute numbers (e.g. articles, citations), qualitative aspects using the modified Hirsch-Index (H-Index), as well as nation’s gross domestic product (GDP), population, infant mortality rates, and development of research over time were taken into account.

## Methods

The methods of this scientometric analysis are based on the previously published Science performance evaluation (SciPE) protocol [8]. As a result, the following search term was systematically created in order to identify articles related to paediatric resuscitation: [Title = (Reanimat* AND (child* OR infant OR newborn OR neon* OR bab* OR pediat* OR paediat*) OR resuscitat* AND (child* OR infant OR newborn OR neon* OR baby OR pediat* OR paediat*) OR Wiederbeleb* AND (Kind OR Säugling OR Neugeboren* OR Kleinkind) OR réanimat* and (Enfant OR néonat* OR nouriss* OR nouveau-né OR postnatal OR pediat* OR paediat* OR bambi* OR bébé OR tout-petit) OR rianimazion* AND (pediat* OR paediat* OR neon* OR bambi*) OR CPR AND (child* OR infant OR newborn OR neon* OR bab* OR pediat* OR paediat*)]. Articles related to resuscitation but not paediatric resuscitation were identified by using the term [Title = (Reanimat* OR resuscitat* OR Wiederbeleb* OR CPR OR réanimat* OR rianimazion* NOT (child* OR infant OR newborn OR neon* OR bab* OR pediat* OR paediat* OR Kind OR Säugling OR Neugeboren* OR Kleinkind OR Enfant OR néonat* OR nouriss* OR nouveau-né OR postnatal OR bambi* OR bébé OR tout-petit OR neon*))]. As several articles were not titled or published in English, the search terms were extended to include results in other common languages (e.g. Spanish, Italian, Russian, French, Japanese, Chinese, Dutch, Polish, Portuguese). Data search included all original research published in the Web of Science core collection (coverage since 1900) for the longest possible observational period (1900–2019). Results from 2020 were excluded to improve comparability, as research articles are frequently postdated. Original articles were identified by selecting the Web of Science category articles and excluding all other categories e.g. reviews or editorials. Data search and extraction was performed on May 24th, 2020. Subsequently, the articles were screened for related data. The defined parameters (e.g. number of publications per nation, citations, data on cooperation, subject areas, and the most publishing authors) were extracted utilizing the analyze results function from the Web of Science and then manually resolved by two independent investigators. In case of disagreements, a third investigator was consulted. All metadata were manually assigned allowing the best possible scientific evidence. Plain visualization techniques were applied using GraphPad Prism and Microsoft Excel. A Padé approximant was used for interpolating the standard curve. The H-Index was used to assess quality of research and expanded e.g. by including nations [9]. Aside from using the H-Index, the total number of articles and citations were taken into account to assess publication performance [8, 9]. Country specific data such as GDP, population, and infant mortality rates were obtained from the Central Intelligence Agency world factbook (Tables 1 and 2) [4, 8].

**Table 1:** Top 20 nations regarding original articles on paediatric resuscitation ranked by H-Index

	Country	H-Index	Articles	Citations	Population [4]	GDP in million [4]	IMR per 1000 [4]
1	United States	70	777	18,863	329,256,465	19,490,000	5.3
2	Canada	36	180	4211	35,881,659	1,774,000	4.3
3	United Kingdom	30	172	3398	65,105,246	2,925,000	4.1
4	Australia	27	141	2671	23,470,145	1,248,000	3.1
5	Norway	24	86	2413	5,372,191	381,200	2.5
6	Spain	17	62	1773	49,331,076	1,778,000	3.2
7	Germany	16	99	1302	80,457,737	4,199,000	3.3
8	Netherlands	16	46	898	17,151,228	924,400	3.5
9	India	15	48	929	1,296,834,042	9,474,000	35.4
10	Italy	14	59	702	62,246,674	2,317,000	3.2
11	Japan	14	36	1008	126,168,156	5,443,000	1.9
12	Austria	13	44	643	8,793,370	441,000	3.3
13	Sweden	12	29	624	10,040,995	518,000	2.6
14	France	11	46	415	67,364,357	2,856,000	3.2
15	New Zealand	10	21	533	4,545,627	189,000	3.5
16	Brazil	9	23	691	208,846,892	3,248,000	15.9
17	South Korea	8	33	398	51,418,097	2,035,000	3.0
18	Switzerland	8	30	291	8,292,809	523,100	3.5
11	China	7	17	170	1,384,688,986	23,210,000	3.3
20	Belgium	6	16	653	11,720,716	529,200	5.3

*GDP* Gross domestic product, *IMR* Infant mortality rate. (Query date: 24.05.2020)

**Table 2:**
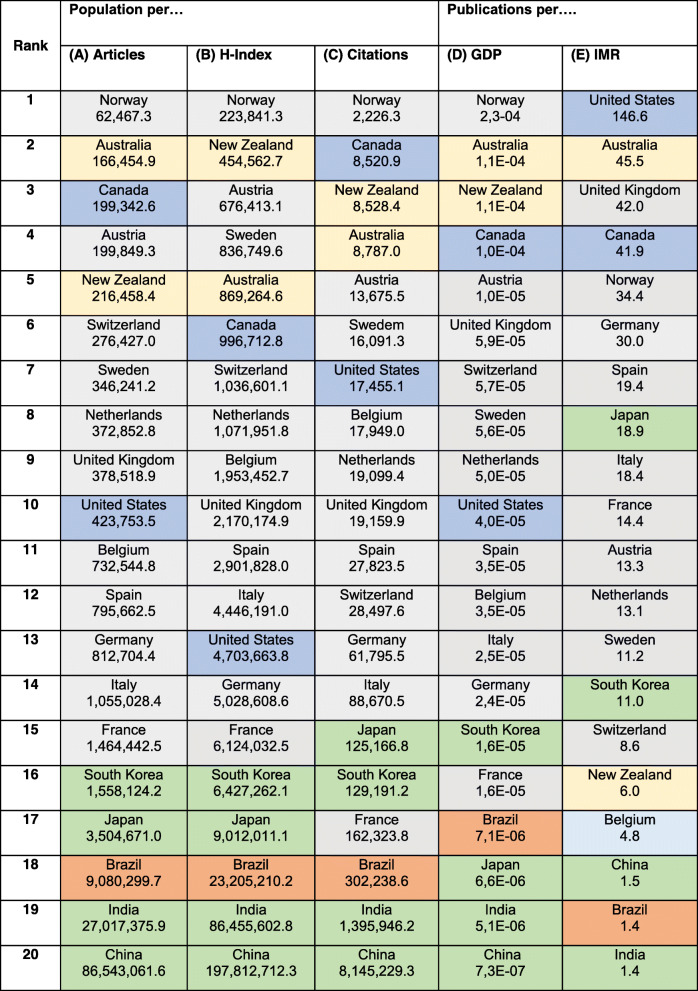
Research on paediatric resuscitation (1900–2019) ranked by scientific output

(A-C): Nations sorted by ratio of population per publications (A), H-Index (B) and citations (C). (C, D): Nations sorted by ratio of publications per GDP (D) as well as infant mortality rate (IMR) (E). Based on Table 1. Colors indicating continent: Asia (green), Europe (grey), North America (dark blue), South America (orange), Oceania (yellow); *GDP* gross domestic product, *IMR* infant mortality rate

## Results

### Global research on paediatric resuscitation

**Fig. 1:**
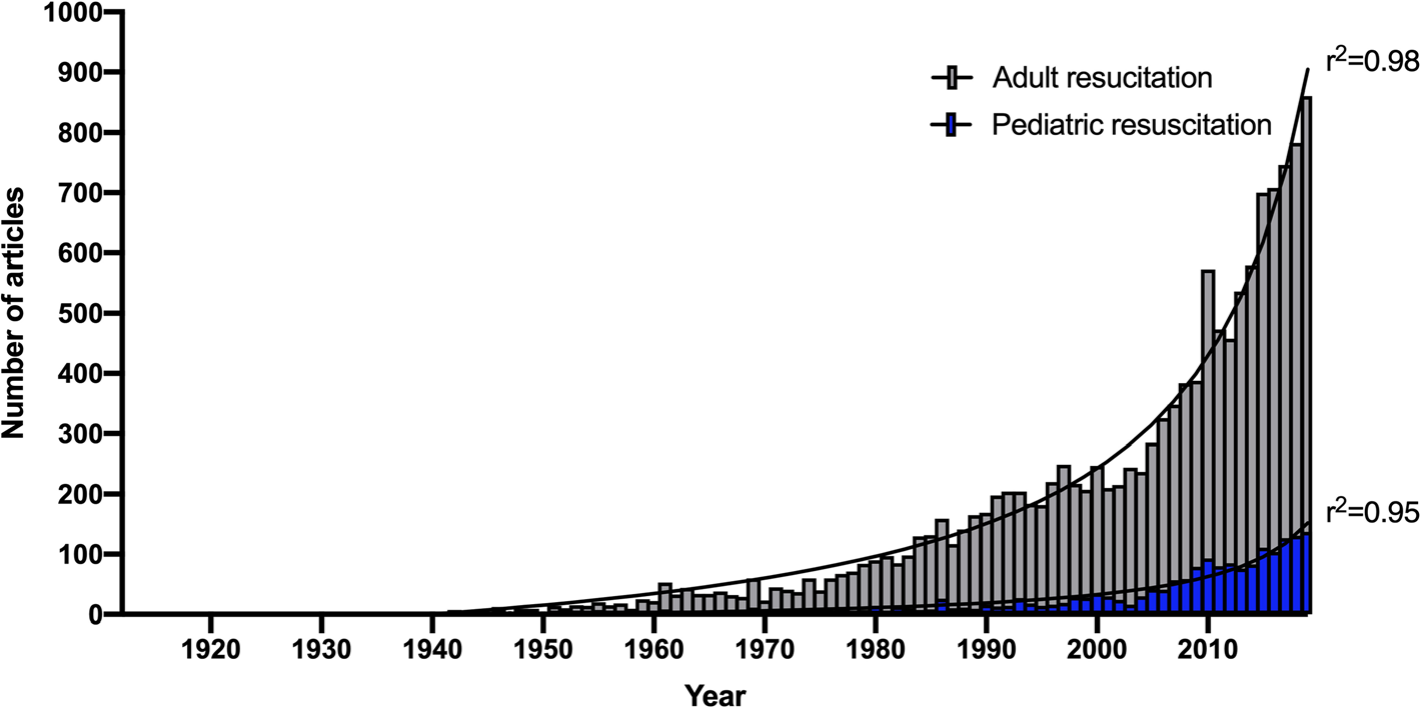
Global research activities on pediatric resuscitation, when compared with research activities on adult resuscitation (1912–2019) and Padé approximant, nonlinear fit. (Query date: 24.05.2020)

**Fig. 2:**
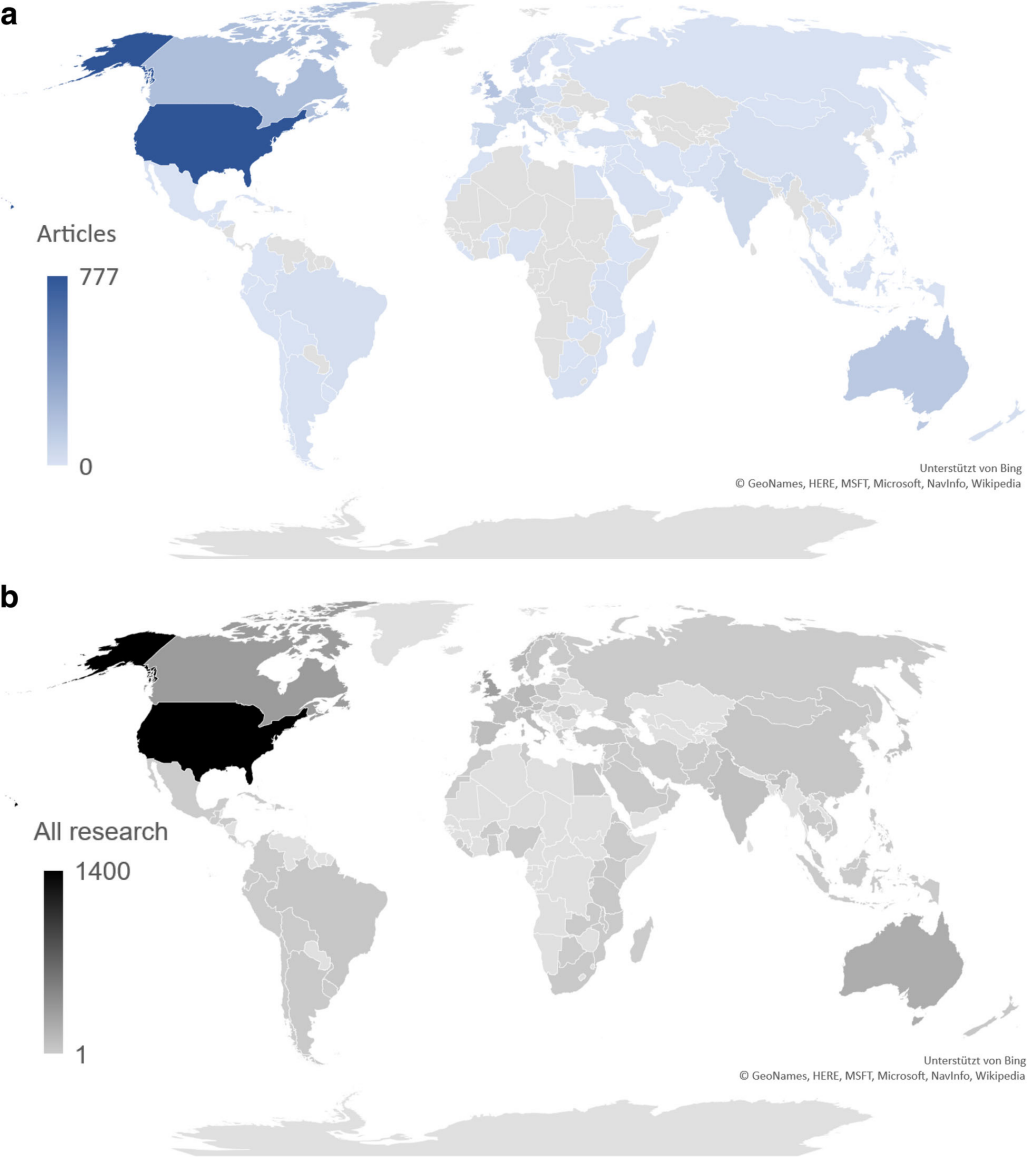
World map illustrating global research on paediatric resuscitation (1900–2019); **a**: Number of original articles per nation. **b**: All published research on paediatric resuscitation, (**b**) (Query date: 24.05.2020); Copyright GeoNames, HERE, MSFT, Microsoft, NavInfo, Wikipedia, supported by Bing

**Fig. 3:**
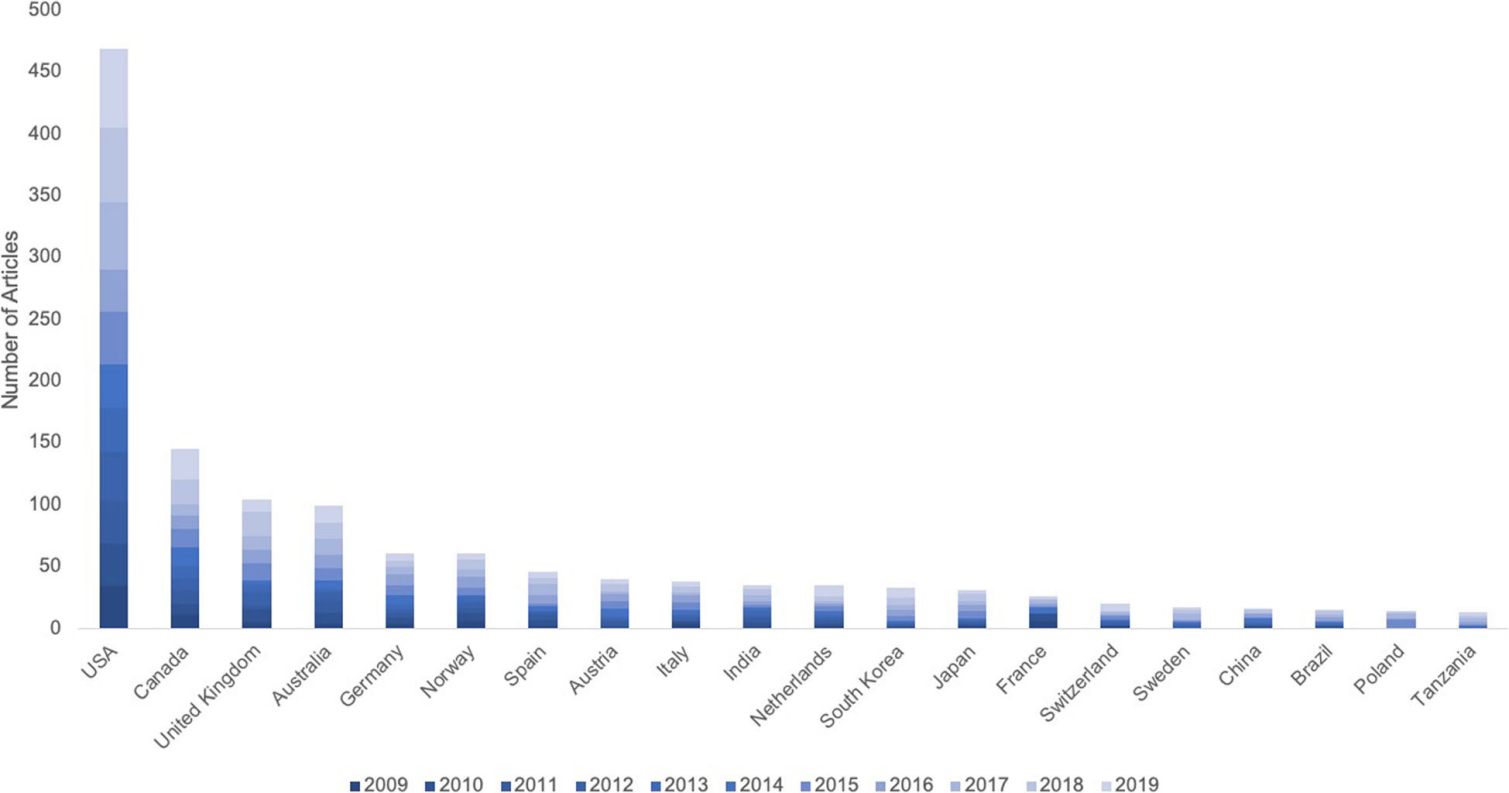
The 20 most publishing nations over the past decade (2010–2019) regarding paediatric resuscitation. USA: United States of America. (Query date: 24.05.2020)

A total of 3468 research items on paediatric resuscitation were published between 1900 and 2019. Out of these, 1896 original articles were identified, that were published by 93 different countries. Notably, there were 26,298 research items on adult resuscitation including 13,749 original articles during the same time period (Figs. 1 and 2). Prior to 1960, there was only little research on both paediatric and adult resuscitation (annually less than 10 original articles). Interestingly, a first publication peak was reached in the 1960s with more than 10 annual original articles on paediatric resuscitation compared to 50 articles on resuscitation of adults. During this time period, the most cited articles concerning paediatric resuscitation focused on hyperbaric ventilation [10, 11] whereas in the 1980s, the topics of the highly cited papers addressed estimation of weight during resuscitation of infants in order to adjust for sufficient medication, survival regarding babies of very low birthweight, and identification of risk factors for sudden infant death [12–14]. For the first time in 2007, 50 annual articles concerning paediatric resuscitation were published. Annual research interest on resuscitation of adults was constantly higher than on paediatric resuscitation but both demonstrate a nearly exponential growth (Fig. 1). Nevertheless, in 2019, 6 times more articles were published on adult resuscitation, when compared to paediatric resuscitation. A total of 1106 original articles, respectively 58% of all research on paediatric resuscitation was published during the last decade (2010–2019; Fig. 3).

### Nations publication quantity

**Fig. 4:**
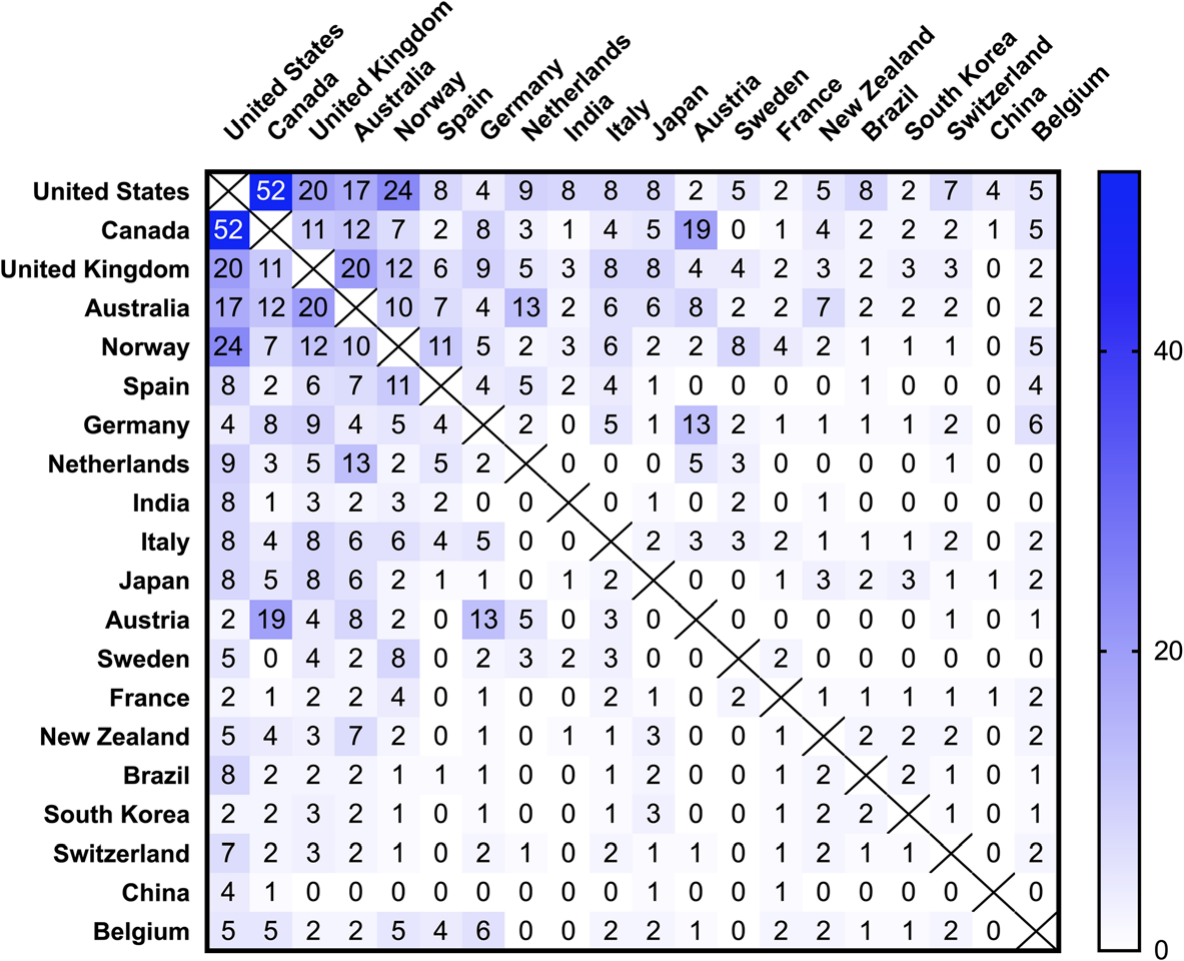
Heat map illustrating international research collaborations on paediatric resuscitation regarding the 20 leading nations (1900–2019), number of cooperation. (Query date: 24.05.20)

The United States is the nation with the highest total number of published original articles, which is more than four times higher than the next-ranked nations Canada and the United Kingdom. Furthermore, Australia, Germany, and Norway belong to the most frequently publishing nations (Table 1). The ranking for the highest number of citations is similar with the lead of the United States, followed by Canada, and the United Kingdom as well as Australia, Germany, Norway, and Spain (Table 1). Analysis of the all-time modified H-Index again identifies the United States in the front position, followed by Canada, and the United Kingdom, Australia, and Norway. If nations publication quantity over the past decade (2010–2019) is compared to the overall total nations’ quantity, the most publishing countries remain almost unchanged (Fig. 3). Notably, Tanzania, Poland, and Brazil were among the 20 most publishing nations during the last decade. In order to identify international research clusters, we analyzed the cooperation patterns of the most frequently publishing countries. In this context, the most productive collaborations were between the United States and Canada (Fig. 4). Additionally, the United States closely cooperated with the United Kingdom, Norway, and Australia. Also, Australia collaborated with the Netherlands. Interestingly, Austria collaborated with Germany and Canada. Detailed heat mapping of international cooperation are depicted in Fig. 4.

### Research on paediatric resuscitation ranked by scientific output

As presented in the SciPE protocol [8], the global research output of the most successful nations was analyzed by ranking the publication performance under consideration of the nation’s population, GDP, and infant mortality rates. In these categories, Europe and North America are again the leading continents. As an individual nation, Norway is ranked first regarding population per article, per modified H-Index, per citations, and per GDP, despite relatively low infant mortality rates. Also, Australia, New Zealand, Canada and Austria occupied leading positions, in synopsis of all parameters. The overall research performance, as an overview of all calculated ratios, is presented in Tables 1 and 2.

### Subject areas

If the allocated Web of Science research categories were analyzed, the 1896 original articles were allocated to 2690 research categories. The majority of 30% was allocated to the category *paediatrics* (801/2690), followed by *emergency medicine* with 15% of all items (390/2690). Other important subject areas were *obstetrics gynecology* (178/2690), *internal medicine* (161/2690), *surgery* (102/2690), *anesthesiology* (91/2690), *cardiac/ cardiovascular system* (76/2690), *public health care* (72/2690), *nursing* (61/2690), and *peripheral vascular disease* (44/2690). Notably, there were allocations to more than 69 further categories.

### Gender analysis of the 100 most frequently publishing authors

Out of the 5862 authors listed in Web of Science for paediatric resuscitation, the 100 most frequently publishing authors were identified and analyzed based on their gender. Of these, 69% were male, 25% were female, and 6% remain unknown.

## Discussion

Research on paediatric resuscitation has grown nearly exponentially between 1900 and 2019 which is in line with various research areas indicating an exponential growth of interdisciplinary research [15–18]. If the leading nations over the past decade are compared to the overall research performance during the complete study period (1900–2019), they remain nearly unchanged indicating consistent leadership of North America and Europe. Out of the top leading nations the United States, Canada, the Netherlands, and South Korea even increased total publication output over the past decade. Thus, leading nations remain the ones with high income, and high educational capacities [4]. However, the efforts on paediatric resuscitation are relatively low when compared to resuscitation of adults and other areas of research [8, 15–18]. Hence, paediatric resuscitation represents a comparatively underrepresented topic. This becomes apparent, when existing evidence gaps are observed including airway management and ventilation, the use of extracorporeal membrane oxygenation as well as therapeutic hypothermia [19]. Potential reasons for the relatively low research performance probably are the rare occurrence rate especially in highly developed countries as well as ethical issues associated with studies in infants and children [4, 7]. However, it has to be emphasized that despite this relatively low research output, resuscitation outcomes have improved considerably in highly developed countries [5, 6]. In this context, it has to be considered that improved outcomes not only depend on research efforts but also on the implementation of this knowledge into everyday practice, specifically in low income settings were infant mortality rates remain highest [4]. If the national research performance on paediatric resuscitation is analyzed based on the absolute numbers of published items and original articles, it is clearly dominated by the United States in respect to both, quality and quantity. This is in line with previous research illustrating the leading position and excellent research possibilities in the United states [15–18, 20]. In contrast, if scientific output is related to population and financial power of the individual nation (e.g. articles, H-index, and citations per population or GDP), Norway is ranked first, whereas the United States is only ranging between ranks 7 to 13. Also, Canada, Australia, New Zealand, and Austria belong to the most important nations regarding research in paediatric resuscitation. In this context, the research efforts of India, Tanzania, and Brazil are of remarkable interest, as these nations are characterized by high infant mortality rates and research efforts regarding other topics in former analyses were relatively low [16–18]. These results bring up the hypothesis that there is special interest of specifically affected nations. Therefore, the lack of research efforts in the majority of heavily affected nations is noteworthy. Sub-Saharan Africa has the highest under-5 years mortality in the world. In 2018 1 out of 13 children died before reaching the age of 5 years. This is 16 times higher than the average ratio in high-income countries [21]. When encountering the underlying causes, improved praenatal care, care at birth, and the postnatal period represent crucial factors. As a result, besides person centred research, population based approaches are needed to improve outcomes. Conclusively, ineffective resuscitation attempts are only a minor reason for high infant mortality rates. The cooperation analyses demonstrated numerous international collaborations. For example, the United States predominantly cooperated with Canada and to a lower extend with the United Kingdom, Norway, and Australia. Besides geographical aspects, the same native language as well as distinctive research efforts of the participating countries may facilitate collaborations. Also, close collaborations across different areas of research induce research activities which becomes apparent, when the subject categories are analyzed. This indicates that paediatric resuscitation is a field affecting different professions e.g. paediatrics, internal medicine, anaesthesiology, and surgery. In addition to international and interprofessional cooperation, close collaboration of nations with high infant mortality rates with leading countries may improve productivity, as demonstrated by the collaboration between the United States and India. Another relevant finding is that the far majority of the 100 leading researchers is male. It has to be mentioned, that multiple approaches on gender analysis have been published [22]. However, to the best of our knowledge, all approaches are limited due to invalid input data, as authors information are often invalid and names are not always predicting authors gender, especially in Asian countries [8, 22, 23]. Hence, for the best possible evidence, authors need to be identified manually, as it was done in the present analysis. Therefore, our finding that only 25% of the top 100 most publishing authors were female might be another indicator for the apparent gender disparities among global research and specifically among research on paediatric resuscitation. This is noteworthy as the leading nations identified in this analysis were also the nations occupying leading positions regarding the educational attainment opportunities sub-index of the global gender gap report ranking [24]. Further analyses for evaluation of gender disparities should summarize higher numbers of authors and should include especially the nations with relatively good development opportunities for female researchers [24].

### Limitations

All citation-based analyses are limited by the quality and quantity of the underlying data. Scientometric studies draw conclusions based on quantitative data. As a result, qualitative statements are only possible to a limited extent. However, careful search and thorough screening of the results were performed by developing precise search terms and using title search. Notably, there may have been articles related to other forms of clinical emergencies that were identified based on our search strategy. In addition, two independent researchers manually screened the identified articles. When analyzing scientific quality using citations and related metrics such as the modified H-Index, self-citations may lead to higher individual modified values but are hardly excludable in everyday practise [25]. Although the present analyses focused on original articles, the complete number of published research is covered by Fig. 2b.

## Conclusion

Research efforts on paediatric resuscitation have increased but the absolute quantity is comparatively small considering the importance of this topic and compared to adults as well as other areas of research. In this context, countries with high infant mortality rates e.g. Tanzania, Brazil, and India are particularly involved but need to be further integrated in international cooperation. Regarding leading researchers, gender disparities are present with a male dominance. As a result, additional efforts are required to overcome gender disparities.

## Data Availability

Please contact author for data requests.

## Abbreviations

SciPE: Science performance evaluation
H-Index: Hirsch-Index
GDP: Gross domestic product
IMR: Infant mortality rate
